# Mechanisms of oxidative stress in human aortic aneurysms — Association with clinical risk factors for atherosclerosis and disease severity

**DOI:** 10.1016/j.ijcard.2013.01.278

**Published:** 2013-10-03

**Authors:** Bartłomiej Guzik, Agnieszka Sagan, Dominik Ludew, Wojciech Mrowiecki, Maciej Chwała, Beata Bujak-Gizycka, Grzegorz Filip, Grzegorz Grudzien, Boguslaw Kapelak, Krzysztof Żmudka, Tomasz Mrowiecki, Jerzy Sadowski, Ryszard Korbut, Tomasz J. Guzik

**Affiliations:** a Translational Medicine Laboratory, Department of Internal and Agricultural Medicine, Jagiellonian University School of Medicine, Cracow, Poland; b Department of Interventional Cardiology, Institute of Cardiology, Jagiellonian University School of Medicine, Cracow, Poland; c Department of Cardiovascular Surgery, Institute of Cardiology, Jagiellonian University School of Medicine, Cracow, Poland; d Department of Vascular Surgery, J. Grande Hospital, Cracow, Poland; e Department of Pharmacology, Jagiellonian University School of Medicine, Cracow, Poland

**Keywords:** Aortic abdominal aneurysm, Oxidant stress, Reactive oxygen species, Inflammation, NAD(P)H oxidase, Superoxide

## Abstract

Aortic abdominal aneurysms (AAA) are important causes of cardiovascular morbidity and mortality. Oxidative stress may link multiple mechanisms of AAA including vascular inflammation and increased metalloproteinase activity. However, the mechanisms of vascular free radical production remain unknown. Accordingly, we aimed to determine sources and molecular regulation of vascular superoxide (O_2_^•^^−^) production in human AAA.

**Methods and results:**

AAA segments and matched non-dilated aortic samples were obtained from 40 subjects undergoing AAA repair. MDA levels (determined by HPLC/MS) were greater in plasma of AAA subjects (n = 16) than in risk factor matched controls (n = 16). Similarly, superoxide production, measured by lucigenin chemiluminescence and dihydroethidium fluorescence, was increased in aneurysmatic segments compared to non-dilated aortic specimens. NADPH oxidases and iNOS are the primary sources of O_2_^•^^−^ in AAA. Xanthine oxidase, mitochondrial oxidases and cyclooxygenase inhibition had minor or no effect. Protein kinase C inhibition had no effect on superoxide production in AAA. NADPH oxidase subunit mRNA levels for p22phox, nox2 and nox5 were significantly increased in AAAs while nox4 mRNA expression was lower. Superoxide production was higher in subjects with increased AAA repair risk Vanzetto score and was significantly associated with smoking, hypercholesterolemia and presence of CAD in AAA cohort. Basal superoxide production and NADPH oxidase activity were correlated to aneurysm size.

**Conclusions:**

Increased expression and activity of NADPH oxidases are important mechanisms underlying oxidative stress in human aortic abdominal aneurysm. Uncoupled iNOS may link oxidative stress to inflammation in AAA. Oxidative stress is related to aneurysm size and major clinical risk factors in AAA patients.

## 1 Introduction

Aortic abdominal aneurysms (AAA) are important causes of cardiovascular morbidity and mortality [1]. They occur in 8% of men after 60 years of age, and are associated with unfavorable prognosis [1]. The mechanisms of AAA are complex and include increased metalloproteinase activity, vascular inflammation, mechanical stress and increased reactive oxygen species (ROS) production in the vessel wall [1]. In spite of many years of research, we are still lacking effective medial therapy for AAA [2]. Therefore, it is imperative to better understand the mechanisms of this important disease and to provide new, specific drug targets for the treatment of AAA.

Recent studies have pointed out, that oxidative stress can provide a link between most known mechanisms of AAA. Reactive oxygen species production is induced by mechanical stress to the vascular wall or by cytokines. Overproduction of ROS may subsequently induce inflammation, matrix metalloproteinase (MMP) activity, smooth muscle cell apoptosis or changes in collagen properties [3]. NADPH oxidases are the major sources of superoxide anion in human and animal vasculature [3,4]. Studies in murine models of AAA demonstrate that NADPH oxidases and the iNOS are also critical for free radical production in aneurysms [5]. Loss of these enzymes prevents the development of AAA, through reduced expression of MMP-2 and MMP-9 in the aortic tissues [5]. For example, p47phox −/− mice, which lack functional NADPH oxidase or mice treated with oxidase inhibitor apocynin are protected from AAA formation [5,6].

Role of local oxidative stress in the pathogenesis of AAA has been much investigated in humans. Some studies have shown that markers of systemic oxidative stress such as malondialdehyde (MDA) are increased in AAA patients [7,8]. Moreover, Miller et al. have demonstrated increased local production of superoxide anion in human aneurysms and suggested that these radicals may come from NADPH oxidases [9,10]. However, the enzymatic and molecular mechanisms of ROS overproduction in human AAA have not been systematically analyzed so far. It is not clear, if the sources are similar to the ones described in animal models. Given vital structural and functional differences between human and mouse aorta, it is an important issue. Moreover, the relationship between clinical risk profile/parameters of the AAA and local vascular oxidative stress remains unclear. Identification of these mechanisms and relationships is critical for the possibility of designing future AAA prevention strategies.

In the present study, using oxidase inhibitors, we identified that NADPH oxidases and iNOS are the primary sources of superoxide anion overproduction in human AAA.

We find, that the expression of NADPH oxidase homologs Nox2 and Nox5 is increased, while Nox4 mRNA is decreased when compared to aorta from non-AAA patients. More importantly, we show for the first time, that superoxide production in aneurysmatic aortic wall is related to overall mortality risk and to aneurysm size, as well as smoking, hypercholesterolemia and the presence of coronary artery disease.

Our study identifies NADPH oxidases and uncoupled iNOS as important drug targets for anti-oxidative strategies in human AAA treatment. Some therapies, already available, can have significant effects on oxidative stress and inflammation in human aneurysms. For example, simvastatin may reduce both ROS production and vascular inflammation in AAA [11]. Inhibition of NADPH oxidases is one of the important pleiotropic effects of statins, however future treatments could be directed more specifically on inhibiting NADPH oxidases or iNOS in AAA [12].

## 2 Materials and methods

### 2.1 Patients and blood vessels

Segments of abdominal aortic aneurysms were obtained during AAA repair surgery at the site of maximal dilatation from 40 patients. In some patients, matching small fragments from non-aneurysmatic wall of the vessel were obtained. Detailed clinical data including major risk factors for atherosclerosis and AAA, as well as intra-operatively determined size of the AAA were recorded at the time of surgery.

Immediately after harvesting, vascular segments were placed in ice-cold Krebs-HEPES buffer and transported to the laboratory for determinations of vascular superoxide production.

In a subgroup of patients, fragments of aortic tissues were saved for cryosections or saved in RNA later solution for subsequent RNA extraction. In a separate group of 5 patients, small segments of aorta were obtained from control subjects undergoing coronary artery bypass graft (CABG) surgery from sites of bypass graft implantation into the aorta and were used for determination or Nox mRNA expression. CABG patients studied were exactly matched for major risk factors to AAA patients from whom samples were obtained for RNA determination. Coronary artery disease (CAD) was defined by clinical history of acute coronary events or positive coronary angiography. Clinical risk factors were categorized as follows: hypercholesterolemia (total plasma cholesterol level > 5 mmol/L); smoking (current or within last 6 months); diabetes (fasting glucose level > 7 mmol/L or HbA_1c_ > 6.5% or current treatment with insulin or oral hypoglycemic agents); and hypertension (blood pressure > 140/90 or current treatment with antihypertensive agents), based on [13]. Mortality risk was assessed in AAA patients using Vanzetto score [14].

Additionally, blood was obtained from matched: 16 AAA and 16 control CAD subjects and was stored for measurements of malondialdehyde (MDA). Ultrasound was used to exclude the presence of AAA in subjects in control group.

Collection of tissues has been approved by the Local Research Ethics Committee and informed consent was obtained. The authors of this manuscript have certified that they comply with the Principles of Ethical Publishing in the International Journal of Cardiology.

### 2.2 Malondialdehyde (MDA) determination

Blood for determination of malondialdehyde (MDA) was collected into sodium citrate and was centrifuged at 1300 RCF (*g*) at 4 °C and plasma was stored at − 80 °C until measurements were performed.

MDA was determined using liquid chromatography with mass spec detection (*HPLC/MS*; *LCQ Finnigan Matt*). A modified technique described in detail by Sim et al. [15] was used. Briefly diluted (10%) samples were incubated with NaOH for 60 min in order to release MDA bound in tissues. The hydrolyzed sample was acidified using 35% (v/v) perchloric acid. Supernatant was then subjected to double extraction using n-hexane. Organic phase was then analyzed using HPLC/MS.

### 2.3 Vascular superoxide production

Superoxide production was measured from intact vessel segments using lucigenin-enhanced chemiluminescence (LGCL; 5 μmol/L), utilizing previously described and validated methods [4,16]. Specificity for superoxide was confirmed by pre-incubation of vascular segments with PEG-SOD (500 IU/mL) or Mn(III)tetrakis(4-benzoic acid)porphyrin chloride (MnTBAP; 25 μmol/L). The maximal activity of NAD(P)H oxidase was measured by adding NADPH (100 μM/L), and O_2_^•^^−^ was detected using 5 μmol/L lucigenin as published before [16,17]. In separate experiments segments of AAA were pre-incubated with various oxidase inhibitors such as apocynin (300 μmol/L), diphenyleneiodonium (DPI; 10 μmol/L); allopurinol (100 μmol/L), rotenone (100 μmol/L), indomethacin (10 μmol/L), nitric oxide synthase (L-NAME, 100 μmol/L), N-(3-aminomethyl) benzylacetamidine (1400W; 2 μmol/L) or chelerythrine (3 μmol/L) [16]. It has to be noted that not all inhibitors used in this study are entirely specific. For example, apocynin — initially thought to be a specific inhibitor of NADPH oxidases possesses also non-specific anti-oxidant properties [18].

### 2.4 Oxidative fluorescent microtopography

In situ superoxide generation was visualized in vascular cryosections (30 μm) with the SOD-inhibitable dihydroethidium (DHE; 2 μmol/L) fluorescence, as described previously [16]. Images were obtained with a BioRad MRC 1024 scanning confocal microscope using the excitation/emission wavelengths 488 nm/610 nm. In each case, paired segments of AAA and AAA pre-incubated with PEG-SOD (500 U/mL) were analyzed in parallel with identical imaging parameters.

### 2.5 Quantitative real-time RT-PCR

RNA was isolated from aortic segments, previously protected with *RNAlater* RNA Stabilization Reagent (QIAGEN), using Qiazol Lysis Reagent (QIAGEN) and purified using RNeasy Lipid Tissue Mini Kit with DNAse digestion. The cDNA synthesized using High-Capacity cDNA Reverse Transcription Kits from 20 ng total RNA was subjected to quantitative PCR using TaqMan Gene Expression Assays (Applied Biosystem) and 7900HT Fast Real-Time PCR System (Applied Biosystem). GAPDH was used as a housekeeping gene for aortic segments using TaqMan Gene Expression Assay. Delta–delta CT was used to calculate mRNA expression for studied Nox homolog subunits. Data are expressed as relative expression (RQ).

### 2.6 Statistical analysis

All data are expressed as mean ± SD with n equal to the number of patients. To test normality of distribution, D'Agostino & Pearson omnibus normality test was employed.

Comparisons between groups of patients or treatments were made using Student t-test or one-way ANOVA, followed by the Student–Newman–Keuls post-hoc test. When indicated, two way ANOVA was implemented. Non-normally distributed variables were analyzed using non-parametric tests such as Mann–Whitney U test or *Kruskal*–*Wallis ANOVA* and are presented as median [10th–90th percentile]. Categorical variables were compared by using chi-square test, as appropriate. Power calculations based on our previous studies of MDA levels suggested that 16 subjects per group would be able to detect a 30% difference in plasma MDA generation with α = 0.05 and power 90%. Similar power calculations of vascular superoxide production showed that 25 subjects per group would be able to detect a 30% difference in vascular superoxide generation with α = 0.05 and power 90%.

Correlation between oxidase activity and aneurysm size was assessed by simple linear regression. Moreover comparison of the relationship between AAA size and NADPH-stimulated superoxide production was performed by comparison of vascular O_2_ generation in groups divided by tertiles of AAA size using a non-parametric *Kruskal*–*Wallis ANOVA* values of p < 0.05 were considered statistically significant. Prism 6.0 for Mac or PASW statistics 18.0 was used for analysis.

## 3 Results

### 3.1 Patient characteristics

Fresh abdominal aortic specimens were obtained from 40 patients undergoing surgical AAA repair. The demographic and clinical characteristics were typical for patients undergoing such surgery, with most prevalent risk factors being male sex, smoking and hypertension (Table 1). AAA patients presented with moderate to large diameters of AAA as determined by pre-operative ultrasound determination (USG) or intra-operatively (Table 2). As expected intraoperative maximal AAA sizes were greater than ones determined by USG although no significant differences were found. Moreover in 31 subjects (77.5%), a thrombus was observed in the lumen of the aneurysm. Blood samples for MDA determination were obtained from 16 subjects undergoing CABG surgery who matched clinical risk factor profile (age, sex, hypertension, smoking, hypercholesterolemia and diabetes) of 16 AAA patients in whom blood was also drawn (Table 1).

### 3.2 Systemic oxidative stress in AAA

**Fig. 1:**
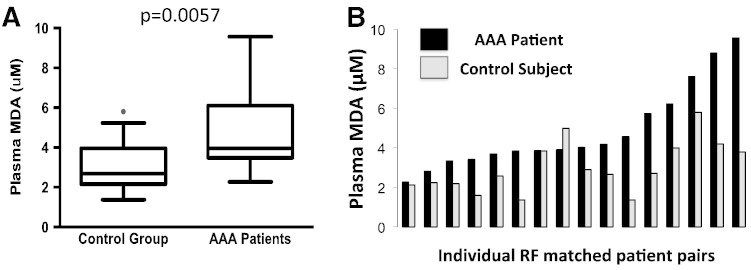
Abdominal aortic aneurysm is associated with systemic oxidative stress. Panel A. Malondialdehyde (MDA) levels in plasma of subjects with AAA and patients without AAA (control group) with similar risk factor profile. Data are presented as median [10th–90th percentile]; (n = 16 in each group) Panel B. Plasma MDA levels in 16 patient pairs, matched exactly for age, sex, and major risk factors for atherosclerosis such as hypertension, hypercholesterolemia, smoking and diabetes.

We measured malondialdehyde (MDA) levels in plasma of 16 AAA and 16 control patients, exactly matched for major clinical risk factors, which have been previously shown to potentially confound oxidative stress measures. Plasma MDA levels were almost two-fold higher in patients with abdominal aortic aneurysms, than in non-AAA CAD group (Fig. 1A). Moreover, detailed analysis of individual patient pairs exactly matched for factors described above, indicated that MDA levels were consistently higher in AAA in all apart from 2 matched pairs (Fig. 1B).

### 3.3 Vascular superoxide production in aortic abdominal aneurysms

**Fig. 2:**
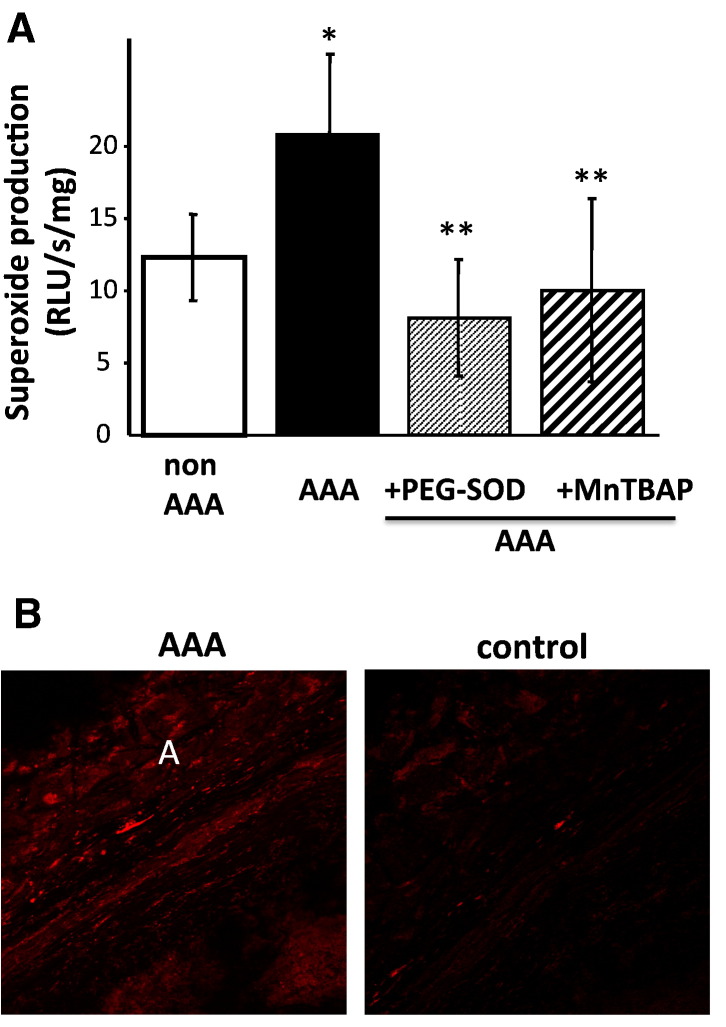
Vascular superoxide production aortic segments from patients with AAA and without AAA. *Panel A*. Basal superoxide production was measured by LGCL (5 μmol/L) in intact segments of aorta obtained from aortic aneurysm at largest diameter point (n = 38) and from non-aneurysmatic segment (non AAA). Superoxide production was expressed in RLU/s/mg dw. Specificity for superoxide was investigated by pre-incubation with PEG-SOD (500 U/mL) or MnTBAP (25 μmol/L) *-p < 0.01 vs. nonAAA; **-p < 0.01 vs no inhibitor. *Panel B.* Histochemical in situ detection of superoxide by DHE staining of abdominal aorta sections obtained from AAA patient. Control sections were incubated with PEG-SOD (500 U/mL). Photograph is representative of n = 5 independent comparisons. A — indicates adventitial side of aortic section.

Basal O_2_^•^^−^ production, determined from freshly-isolated aortic abdominal aneurysms, by LGCL (5 μmol/L), was greater in aneurysmatic segment (isolated from the site maximal dilatation) than in non-aneurysmatic segments of the aorta obtained from the same patient (Fig. 2A). For reference, we have also measured O_2_^•^^−^ production from segments of aorta isolated from non AAA patients, obtained during CABG at the sites of implantation of venous bypass grafts, and found it to be comparable to the level of superoxide produced by non-aneurysmatic areas of aorta in AAA subjects (11.5; SD 3.1 RLU/s/mg dw; n = 5).

Moreover, in situ dihydroethidium staining showed that O_2_^•^^−^ production was observed in all vascular layers of aortic wall from AAA patients, and that it appeared to be the highest at the adventitial side of aneurysmatic aortic wall (Fig. 2B).

### 3.4 Sources of superoxide production in human abdominal aneurysms

**Fig. 3:**
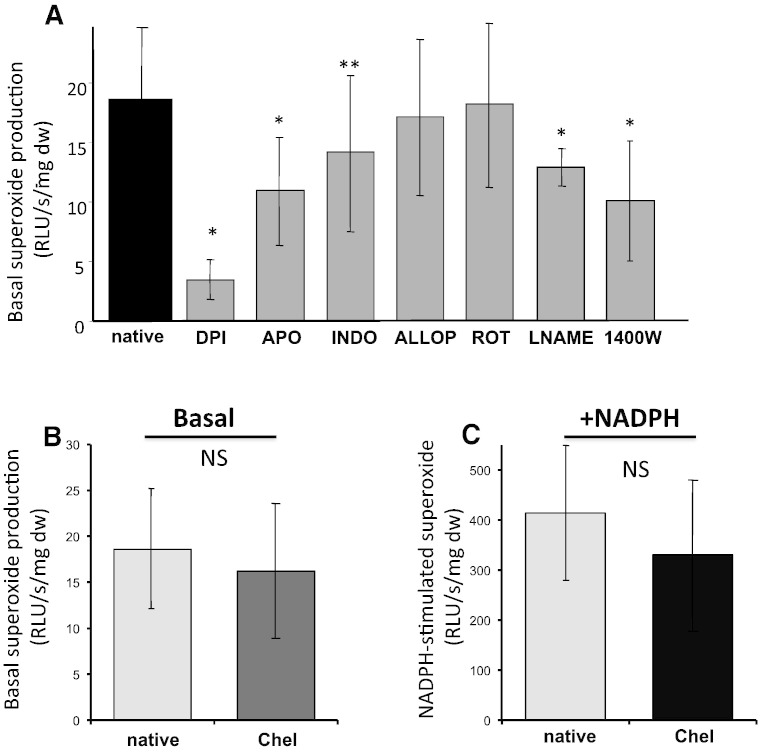
Sources of vascular superoxide anion in human abdominal aortic aneurysms. Panel A. Superoxide production was determined by LGCL (5 μmol/L) in intact aortic segments (n = 6), following a 30 minute incubation with various oxidase inhibitors: NADPH oxidase (apocynin, 300 μmol/L, not entirely specific), diphenyleneiodonium (DPI; 10 μmol/L); xanthine oxidase (allopurinol, 100 μmol/L), mitochondrial oxidases (rotenone, 100 μmol/L), cyclooxygenase (indomethacin, 10 μmol/L), nitric oxide synthase (L-NAME, 100 μmol/L) or iNOS (1400W, 2 μmol/L). Panel B. Effects of protein kinase C inhibition on superoxide production in AAA. Basal (left graph) and NADPH (100 μmol/L) stimulated (right graph) superoxide production was measured in intact segments of AAA using LGCL (5 μmol/L). Adjacent segments were pre-incubated for 30 min with or without an inhibitor and luminescence was measured in the absence/presence of protein kinase C inhibitor chelerythrine (Chel) at 3 μmol/L.

We next investigated the enzymatic sources of O_2_^•^^−^ production in intact segments of human AAA aorta using a range of oxidase inhibitors (Fig. 3A). The greatest inhibition of O_2_^•^^−^ production in aortic segments was caused by NADPH oxidase inhibitors (diphenyliodonium and apocynin). Allopurinol, a xanthine oxidase inhibitor, did not decrease vascular O_2_^•^^−^ production. Similarly, oxypurinol, which was used in a subset of experiments as an active metabolite of allopurinol, did not have any significant effect (data not shown). Interestingly, an additional 20% inhibition of O_2_^•^^−^ production in AAA aortas was observed in response to indomethacin, the cyclooxygenase inhibitor.

Notably, significant effects were observed upon pre-incubation of vascular segments with nitric oxide synthase inhibitors. Both L-NAME (ca. 30% reduction) and 1400W (iNOS specific inhibitor; ca. 40% reduction) resulted in a very significant reduction of basal O_2_^•^^−^ production in aortic abdominal aneurysms, indicating an involvement of iNOS in superoxide generation in AAA.

These studies have identified NADPH oxidases as principal sources of O_2_^•^^−^ production in AAA. As vascular NADPH oxidases are commonly regulated by protein kinase C, we next studied the role of protein kinase C (PKC) in modulating vascular O_2_^•^^−^ production. Inhibition of PKC by chelerythrine did not affect basal vascular O_2_^•^^−^ production in AAA (Fig. 3B). We next studied effects of chelerythrine on NADPH-stimulated oxidase activity in AAA aortas, but similarly to basal O_2_^•^^−^ production we found no effect of PKC inhibition on NADPH oxidase activity in AAA (Fig. 3C).

Taken together, these findings suggest that NAD(P)H oxidases are the major sources of superoxide in human AAA, with an additional contribution of cyclooxygenases and iNOS. Surprisingly, increased NAD(P)H oxidase activity in AAA is not regulated by PKC.

### 3.5 NAD(P)H oxidase subunits in human abdominal aneurysms

**Fig. 4:**
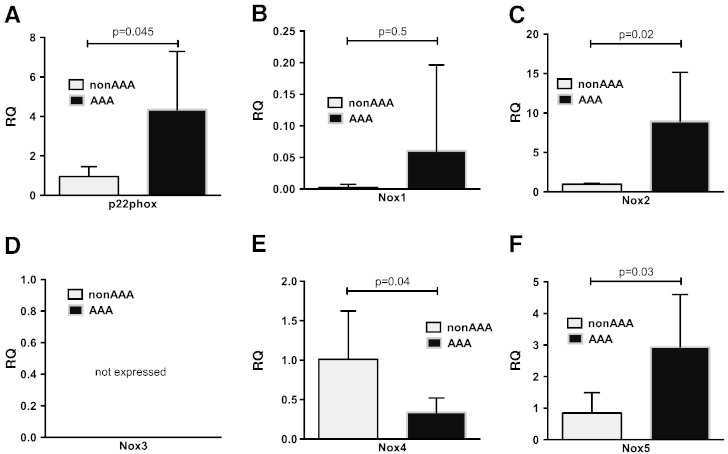
Molecular composition of NAD(P)H oxidases in human abdominal aortic aneurysms (n = 5) and aortic segments from risk-factor matched CABG patients (n = 5). Quantitative RT-PCR (TaqMan) of NADPH oxidase subunits p22phox (panel A), nox1 (panel B), nox2 (panel C), nox3 (panel D), nox4 (panel E) and nox5 (panel F) was determined using 20 ng of total RNA. ddCT was used for calculation with GAPDH as a reference gene. Data are expressed as mean relative expression (RQ) ± SD; *-p < 0.05 vs. non AAA; **-p = 0.05; NS — non significant; NE — not expressed.

Since NAD(P)H oxidases appear to play the principal role in superoxide production by human abdominal aortic aneurysms, we next sought to evaluate the NAD(P)H oxidase membrane subunit mRNA expression in aortic AAA extracts. NAD(P)H oxidase subunit expression (nox1, nox2, nox3, nox4, nox5 and p22phox) was measured using TaqMan quantitative real-time fluorescent RT-PCR. We found that p22*^phox^* and nox2 mRNA levels were dramatically increased in AAA segments when compared to control aortas (Fig. 4). Interestingly a significantly higher expression of Nox5 was also observed. Very low expression of Nox1 was found in only 2 out of 5 studied aortic extracts, while it was not observed in control vessels at all. However due to very low level of expression this difference did not reach statistical significance. Nox3 mRNA was not detectable in any of the studied samples.

Interestingly we observed that Nox4 expression was lower in AAA aortic extracts than in control samples (Fig. 4).

### 3.6 Superoxide production in AAA and risk profile

We next sought to determine the relationships between risk factors for abdominal aneurysms and atherosclerosis with vascular superoxide production in AAA. We therefore analyzed the difference in superoxide production from AAA aortic segments between patients with low and high risk profiles. Risk profile was determined using Vanzetto scale, which is commonly used in the clinical practice to assess surgical risk in patients with aortic abdominal aneurysms. Vanzetto scale uses following clinical parameters to assess risk: age > 70 years; coronary artery disease, Q or ST changes in the ECG, co-existence of congestive heart failure, hypertension or diabetes with left ventricular hypertrophy.

**Fig. 5:**
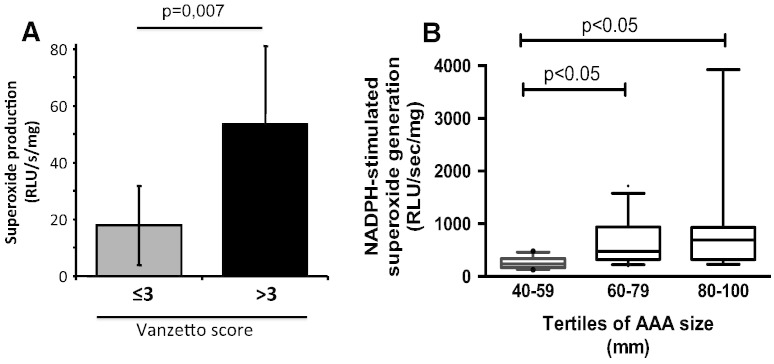
Relationship of clinical characteristics to vascular superoxide generation from AAA. Panel A. Superoxide production was determined by LGCL (5 μmol/L) in patients with Vanzetto score ≤ 3 and > 3. Data are expressed as mean ± SD. Panel B. Relationships between intra-operatively determined AAA size and NADPH-stimulated superoxide production was (n = 30) determined in AAA segments using LGCL (5 μmol/L) at the site of largest dilatation. Data is analyzed for tertiles of AAA size and non-parametric Kruskal–Wallis ANOVA (p = 0.0018) with post-hoc multiple comparisons was performed.

We observed that subjects who presented with Vanzetto score > 3 showed more than tripled superoxide production in their aortic segments (Fig. 5A), indicating a clear relationship between vascular superoxide production and risk in AAA patients undergoing AAA repair surgery.

We next analyzed the effects of individual risk factors on O_2_^•^^−^ production in segments of AAA. Smoking, hypercholesterolemia and presence of coronary artery disease were most strongly associated with vascular superoxide production (Table 3). It was surprising that male sex, which is a very strong risk factor for AAA was not significantly associated with O_2_^•^^−^ production in AAA. On the contrary, it appeared that women showed greater tendency towards increased superoxide production than men. It should be however remembered that majority of subjects in AAA group were male and hypertensive (Table 1), thus the analysis of the effects of sex on superoxide production in AAA may not have enough statistical power to draw meaningful conclusions regarding effects of male sex or hypertension.

### 3.7 Aneurysm size and NADPH oxidase activity

We next studied the relationship between vascular basal and NADPH-stimulated O_2_^•^^−^ production from AAA and aneurysm size determined intra-operatively. We observed statistically significant positive correlations between these parameters, although correlation between aneurysm size and NADPH-stimulated O_2_^•^^−^ production (as a measure of NADPH oxidase activity) was stronger than with basal superoxide production (R = 0.5; p < 0.01 vs. R = 0.3; p < 0.05 using the Spearman correlation test). As aneurysm size was not entirely continuous variable, we grouped the patients in this figure according to tertiles of AAA size and compared NADPH-stimulated superoxide values across the tertiles by using a multiple comparisons Kruskal–Wallis ANOVA test (p = 0.0018) (Fig. 5B).

## 4 Discussion

Increased vascular oxidative stress is a feature of several vascular diseases. In the present study we show that human abdominal aortic aneurysms are associated with systemic oxidative stress and increased local O_2_^•^^−^ production within the aneurysm. Using pharmacological inhibitors we demonstrate, that the NADPH oxidases and iNOS are the major sources of superoxide, and that cyclooxygenases may also modestly contribute to O_2_^•^^−^ production in AAA. NADPH oxidase activity measured as NADPH-stimulated superoxide anion production is correlated to the size of aneurysm. We also found that the NADPH oxidase subunits p22phox, nox2 and nox5 are increased in AAA, while nox4 is reduced. Interestingly, we did not find the role for protein kinase C in the regulation of vascular oxidases in AAA, in contrast to coronary artery disease or diabetes [19]. Moreover, we describe that superoxide production in AAA is significantly associated with several clinical risk factors for atherosclerosis and that it is significantly higher in AAA segments obtained from patients with higher peri-operative risk score.

Previous studies have suggested that reactive oxygen species production is increased in human AAA [9,10]. Here, we confirm, that O_2_^•^^−^ production is increased in AAA, when compared to non-aneurysmatic regions of the aorta [10]. However, the difference between the two, observed in the present study is smaller than previously reported by Miller et al. [10]. We studied much larger patient population, but this difference may be related to the increasing use of statins and other medications known to reduce reactive oxygen species production in patients at high cardiovascular risk. Indeed, 60% of our subjects used statins, while the statin use was not reported in the initial paper [10].

Our study further extends these initial observations, by relating vascular AAA superoxide production to clinical characteristics of the patients. We have observed that AAA vascular superoxide production is much higher in patients with high overall mortality risk assessed by Vanzetto score [14]. This finding is important as it shows potential clinical importance of increased oxidative stress in AAA, although it needs to be further prospectively evaluated in a larger cohort of patients. We used Vanzetto score as one of the comprehensive risk assessment scores utilized in AAA patients. Recent studies clearly show that results obtained with Vanzetto score are strongly correlated with risk stratification using other clinical risk stratification scales such as Eagle score, Glasgow aneurysm score, Leiden score or the modified Leiden score [14]. The clinical importance of our observations is also related to the fact that current treatment options for AAA are very limited. We are able to monitor the progressive increase of AAA diameter, until the criteria for surgical repair are fulfilled or the aneurysm ruptures. We are unable to effectively slow down of prevent the development of the disease [1,2].

We then analyzed the relationships between major individual AAA risk factors (such as hypertension, smoking, male sex, dyslipidemia and co-existing CAD) and vascular superoxide production. Previous studies, including our own, have clearly shown that superoxide production in human conduit vessels, such as saphenous veins, radial arteries or human mammary arteries is closely linked to patient's age and major risk factors for atherosclerosis [4,20–22]. This has not been addressed in AAA patients before. We have found, that smoking, hypercholesterolemia and co-existence of CAD are significantly associated with increased O_2_^•^^−^ production from the AAA wall. This is interesting, in particular in relation to smoking, which is a very strong risk factor for the development of AAA. This finding could provide a mechanistic link between smoking, increased local vascular oxidative stress and the development of aneurysm.

Interestingly we did not observe significantly increased AAA superoxide production in relation to hypertension or male sex, which are the most important risk factors for the AAA. At the same time we have now observed the effect of dyslipidemia, which appears to be an emerging risk factor for AAA [23]. Further explanation of these relationships will require larger cohort studies focused on the role of hypertension and dyslipidemia in oxidative stress related to. Due to relatively small sample size we did not perform multivariate analysis of the effect of risk factors on vascular superoxide production, which should be a subject for larger clinical studies. It should however be noted, that in our previous studies of human vasculature, we have often not seen associations of vascular ROS with hypertension in multivariate analysis even in much larger studies [4]. This may be attributed to the effect of treatments for hypertension, which are known to reduce ROS production [24].

While the oxidative stress has been defined in AAA before, the enzymatic sources of ROS had remained unclear, making it difficult to design specific interventions targeting oxidative stress. To address this issue, we measured superoxide production following pre-incubation with several oxidase inhibitors. Similar studies in conduit vessels of patients undergoing coronary artery bypass grafts [3,25,26] or human varicose veins [27] have shown that NADPH oxidases and eNOS and in some cases xanthine oxidases, cyclooxygenases or mitochondrial oxidases are the most important sources of superoxide anion in human vessels[3,25,26]. Therefore in the present study we used inhibitors of these oxidases, and we have found that NADPH oxidases, iNOS and to the lesser extent cyclooxygenases contribute to net superoxide production in sections of human AAA. This is in agreement with previous studies in animal models of AAA, which had shown that NADPH oxidases and iNOS are predominant sources of superoxide anion in AAA [5,6]. Moreover, model studies using NADPH oxidase knockout had shown that loss of functional NADPH oxidase inhibits generation and progression of AAA [5,6].

In the present study, we demonstrate for the first time that aneurysm size is closely correlated to NADPH oxidase activity, which provides further argument for the role of this enzyme system in the progression and development of the disease. Increased superoxide production can result in the activation of metaloproteinases such as MMP-2 and MMP9, which are critical for the development of AAA [5,6]. Moreover O_2_^•^^−^ modulates inflammatory reactions and can enhance leukocyte recruitment, which contribute to AAA progression [1,5,6]. T is now important to unravel, how are the NADPH oxidases activated in AAA. These enzymes have been shown to be regulated by risk factors for atherosclerosis as well as numerous genetic, endocrine and paracrine factors [28]. Relationships to similar clinical features, which we observed in AAA, can indicate, that activation and increased expression of NADPH oxidases in aneurysmatic vessel wall can be greatly affected by “simple” clinical risk factors of AAA development.

Having identified NADPH oxidases as predominant sources of superoxide anion in AAA, we characterized the molecular composition of these enzymes in AAA. We identified, that nox2 and nox5 are the predominant NADPH oxidase homologs expressed in aneurysmatic vessel wall.

Oxidative stress is closely linked to inflammation in a variety of cardiovascular disorders, including atherosclerosis, myocardial infarction, atrial fibrillation [29] and others [30]. Therefore anti-oxidant based interventions are considered to be potentially important for treatment of number of inflammatory and cardiovascular disorders [31]. Increase of NADPH oxidase activity and expression of Nox2 and p22phox which we observed in AAA, may be in part related to inflammatory infiltration of the vessel wall, as we have previously observed in atherosclerotic human coronary arteries [20]. However, dihydroethidium staining of the AAA wall indicates that superoxide production is observed throughout the aneurysmatic aortic wall, and appears to be the strongest at the adventitial side. We have performed additional experiments in which we measured LGCL from AAA section in which adventitia or intima was facing the detector and repetitively we observed that superoxide detection was particularly visible from adventitial side (data not shown). Future studies using immunohistochemistry and immunofluorescence are warranted to identify cellular sources of free radicals in AAA.

The observation that nitric oxide synthases contribute to vascular wall oxidative stress in AAA is very interesting. To our surprise we noted that iNOS specific inhibitor 1400W caused greater degree of inhibition of superoxide production in AAA segments than pan-NOS inhibitor L-NAME. This, however, may be an indirect suggestion that in AAA other NOS enzymes (eNOS and nNOS) are net contributors of NO rather than superoxide, while iNOS is a net producer of superoxide anion in AAA. This is related to nitric oxide synthase biology which, depending on conditions such as tetrahydrobiopterin or l-arginine availability, can produce either nitric oxide or superoxide [32,33]. Uncoupled NOS enzyme transfers electrons directly onto the molecular oxygen, leading to the generation of superoxide anion rather than nitric oxide. Uncoupling of vascular NOS can be functionally determined using NOS inhibitors such as L-NAME due to the rapid interaction between nitric oxide and superoxide [34]. If NOS produced nitric oxide, its inhibition would lead to increase of superoxide bioavailability. If, in turn, NOS produced net superoxide rather than NO, inhibition of NOS would lead to decrease of superoxide released from the tissue [34]. Therefore the difference between degree of O_2_^•^^−^ inhibition by L-NAME and 1400W clearly suggests that iNOS is a superoxide producing enzyme in AAA, while other NOS might still be able to produce nitric oxide. The observation of the dysfunction of iNOS, could indicate that tetrahydrobiopterin supplementation, could be tested as a possible strategy to limit oxidative stress in AAA.

While our study has focused on oxidant mechanisms, it is possible that impaired anti-oxidant capacity may also play an important role in oxidative stress in human AAA. Several studies in both animal models and humans point to an imbalance and dysfunction of anti-oxidant enzymes in aortic abdominal aneurysms, in part in relation to clinical risk factors such as hypertension [35,36]. SOD and other anti-oxidant mechanisms have been implicated in a number of cardiovascular diseases [37,38]. We have also shown expression of superoxide dismutases in human conduit vessels, although the role of these enzymes seemed moderate when compared to superoxide generating enzymes [39].

While our study focused on local ROS production in the aneurysm, we show here that these local events have systemic consequences, which can be observed in plasma. Using HPLC with mass-spectrometry detection we confirmed previous observations, that malondialdehyde levels, as a measure of systemic oxidant stress are significantly higher in the plasma of AAA subjects [8]. The major advantage of the approach presented in this paper is that we carefully matched patients for age, sex, and major clinical risk factors, which are known to affect vascular and systemic oxidative stress. Such approach of matching subjects is particularly valuable in relation to variables such as superoxide production, which are affected by common risk factors for atherosclerosis and was previously successfully applied [16]. Here we show individual patient pairs to demonstrate the very strong difference. This shows clearly, that systemic oxidative stress was very significantly increased in subjects with AAA. The mechanism for this increase may be related to systemic inflammatory reaction in AAA, or even more likely, to the generation of reactive oxygen species locally in the aneurysmatic aorta. MDA levels might be increased by aneurysm repair surgery itself [8], however it was not the case in our study, as blood was drawn prior to surgery.

In the present study we demonstrated, for the first time, a significant correlation between NADPH-stimulated vascular superoxide anion production in AAA segments and intra-operatively determined aneurysm size. Previous studies have identified relationships between aneurysm size and systemic oxidative stress, measured by MDA in human aneurysms [8]. Demonstration that NADPH oxidase activity is correlated with aneurysm size may further indicate the importance of this enzyme in aneurysm progression and disease pathogenesis. More importantly, it also shows the clinical impact of increased NADPH oxidase activity in AAA, which indicates that NADPH oxidases may be valuable drug targets in human AAA.

## 5 Study limitations

Our study has certain limitations. Firstly, the sample size, although larger than in the previous studies may not be sufficient for desired multivariate epidemiological analysis of the effects of larger number of clinical factors on oxidative stress parameters. Our study is so far the largest to our knowledge to systematically quantify vascular superoxide production in AAA samples in relation to clinical factors. However, while we have pre-determined statistical power of comparisons, it cannot be excluded that lack of difference in vascular superoxide production in relation to hypertension or male sex may be related to small number of patients without these risk factors in our study population.

Our study focused solely on pro-oxidant mechanisms of oxidative stress while the role of antioxidant systems in pathogenesis of AAA has also been described. As emphasized in Materials and methods section, we used pharmacological inhibitor approach to determine the relative contribution of various vascular oxidases to AAA superoxide generation. While this approach is commonly employed, we must remember that not all inhibitors are entirely specific. For example apocynin, initially thought to be a specific inhibitor of NADPH oxidases also have non-specific anti-oxidant properties [18]. In the present study, we did not analyze individual layers of the aneurysm wall, in relation to their contribution to superoxide production; therefore it is impossible to accurately determine the contribution of intima, media or adventitia to vascular superoxide production. Infiltrating inflammatory cells may also contribute to oxidative stress in the tissue, but difficulty in distinguishing these cells as a source of superoxide anion is inherent to the approach of whole vessel studies used here. On the other hand, measurements in human tissue have particular translational value, as they give a direct insight into human disease, as opposed to animal or cell culture models.

## 6 Conclusions

In conclusion, we have shown, that superoxide anion is widely produced in human abdominal aortic aneurysms, and that it is particularly increased in adventitial surface of AAA. Its production is significantly related to aneurysm size, clinical risk factors for AAA and atherosclerosis and is more than tripled in patients with highest mortality risk. Nox2 and Nox5 based NADPH oxidases appear to be the predominant contributors to oxidative stress in human AAA, while iNOS may also contribute.

This may identify NADPH oxidases and uncoupled iNOS, as potentially important drug targets for the treatment of oxidative stress in aortic abdominal aneurysms. The search for novel pharmacological therapies is particularly important, as we now rely almost exclusively on invasive and surgical mechanical repair techniques [2]. Having said that, our study clearly indicates, that intervention into classical risk factors for AAA should be the first step in treating oxidative stress in AAA.

## Figures and Tables

**Table 1:** Clinical characteristics of patients from whom aortic specimens and/or plasma were obtained. CAD — coronary artery disease; BMI — body mass index; PAD — peripheral arterial disease; TIA — transient ischemic attacks; ACE-I — Angiotensin Converting Enzyme Inhibitor.

	Control group	AAA	p value
N	16	40	
Age (mean ± SD)^a^	64.6 ± 6.9	65.7 ± 6.7	0.70
Sex (M:F)^a^	12:4	36:4	0.14

*Risk factors*			
Smoking (n; %)^a^	12 (75%)	28 (70%)	0.70
Hypertension (n; %)^a^	12 (75%)	31 (77.5%)	0.84
Diabetes (n; %)^a^	1 (6.2%)	3 (7.5%)	0.87
Hypercholesterolemia (n; %)^a^	10 (62.5%)	26 (65%)	0.86
BMI	29.7 ± 5.2	26.9 ± 3.3	0.60
Cholesterol (mmol/L; mean ± SD)	6.1 ± 1.5	6.3 ± 1.8	0.50
CAD (n; %)^a^	16 (100%)	36 (90%)	0.18
PAD, TIA (n; %)	5 (31%)	14 (35%)	0.78

*Main medications*			
Aspirin	12 (75%)	27 (68%)	0.58
ACE-I	6 (37%)	25 (62.5%)	0.07
β-Blocker	9 (56%)	14 (35%)	0.14
Statin	10 (62.5%)	24 (60%)	0.86

a Indicates factors which were exactly matched for the comparison of MDA between patients (data shown in Fig. 1).

**Table 2:** Morphologic characteristics of studied aneurysms.

	Parameter		Average ± SD
Ultrasound assessment	Maximal AAA aortic size (L3)	mm	64.5 ± 17.8
	Sub-diaphragmatic aortic size (L1)	mm	26.0 ± 4.1
	Enlargement ratio (L3/L1)		2.4 ± 0.6
Intraoperative assessment	Maximal size	mm	69.2 ± 21.9
	AAA with thrombus		77.5%

**Table 3:** Major risk factors for atherosclerosis and AAA in studied population in relation to superoxide production. Data are shown as univariate analysis.

Risk factor	Basal superoxide production (RLU/s/mg dry weight)		p value
	With RF	Without RF	
Male sex	8.44 ± 2.55	29.82 ± 14.16	0.22
Hypertension	31.55 ± 12.73	22.56 ± 10.2	0.45
Smoking	37.08 ± 13.89	13.38 ± 2.75	0.03
Hypercholesterolemia	40.36 ± 16.23	13.89 ± 3.28	0.013
Coronary artery disease	41.79 ± 15.91	14.05 ± 4.71	0.037
